# Chimeric RNA landscape in the placenta: A transcriptomic analysis revealing novel diagnostic biomarkers forpreeclampsia

**DOI:** 10.1016/j.gendis.2024.101242

**Published:** 2024-02-09

**Authors:** Jingjing Xu, Xinrui Shi, Shitong Lin, Sandeep Singh, Samuel Haddox, Christopher Phung, Tommy Manley, Ningxi Huang, Peng Wu, Hui Li

**Affiliations:** a Department of Obstetrics and Gynecology, Tongji Hospital, Tongji Medical College, Huazhong University of Science and Technology, Wuhan, Hubei 430030, China; b Department of Biochemistry and Molecular Genetics, University of Virginia, Charlottesville, VA 22908, USA; c Department of Obstetrics and Gynecology, Union Hospital, Tongji Medical College, Huazhong University of Science and Technology, Wuhan, Hubei 430030, China; d Cancer Biology Research Center (Key Laboratory of the Ministry of Education), Tongji Hospital, Tongji Medical College, Huazhong University of Science and Technology, Wuhan, Hubei 430030, China; e Department of Pathology, University of Virginia, Charlottesville, VA 22908, USA; f Department of Biomedical Engineering, University of Virginia, Charlottesville, VA 22908, USA; g Computational Toxicology Facility, CSIR-Indian Institute of Toxicology Research, Lucknow, Uttar Pradesh, 226001, India

Chimeric RNAs, hybrid transcripts containing exons from two originally separate genes, have been extensively studied in various cancer types and non-cancerous tissues.^1^^,^^2^ The hybrid transcripts can arise through different mechanisms, such as chromosomal rearrangements, trans-splicing events, or read-through transcription. The investigation into chimeric RNA landscape across various cancer types as well as in normal physiology processes has highlighted their potential roles in regulating gene expression and cellular functions. However, the specific landscape and functional relevance of chimeric RNAs in pregnancy-related conditions, such as gestational complications, fetal growth abnormalities, or preterm birth, are still not fully understood. Preeclampsia is one of the most high-risk pregnancy complications that lack early diagnosis and precise treatment.^3^^,^^4^ Here, we explored chimeric RNAs in placenta tissues and their potential clinical implications in preeclampsia. Firstly, we identified 272 placenta-specific chimeric RNAs by analyzing the placenta RNA-seq data. We then validated the candidate chimeric RNAs using a combination of bioinformatic analyses and experimental validations. To further explore their potential as biomarkers for preeclampsia, we conducted bioinformatic analysis on the expression of candidate chimeric RNAs in placenta RNA-seq data, comparing preeclampsia samples with age-matched healthy pregnancies. We discovered three chimeric RNAs that exhibited a higher or specific expression pattern in preeclampsia placental samples. We then validated their expression in clinical samples of preeclampsia placentas and healthy controls. *PGBD2-SZT2* chimeric RNA was discovered as a potential diagnostic biomarker due to its significantly higher expression in preeclampsia placentas. These findings uncover a new repertoire of pregnancy-specific chimeric RNAs and highlight their potential as novel diagnostic biomarkers or therapeutic targets for preeclampsia.

To explore the chimeric RNAs in the placenta, we identified the chimeric RNAs using the EricScript software from the public database GSE143953 which includes the raw RNA sequencing data of eight placenta tissues (Fig. 1A). To further discover the chimeric RNAs especially expressed in the placenta, we filtered out the chimeric RNAs which have been identified in 54 normal tissues from the GTEx project. Ultimately, we identified 272 placenta-specific chimeric RNAs (Fig. 1B). According to the localization of the parental genes, we categorized them into inter-chromosomal, intra-chromosomal, and read-through chimeric RNAs. Among the placenta-specific chimeric RNAs, 96% are classified into inter-chromosomal or intra-chromosomal chimeric RNAs, while only 4% of the placenta-specific chimeric RNAs are read-through chimeric RNAs (Fig. S1A). We also classified them into “E/E”, “E/M”, “M/E”, or “M/M” based on the location of junction sites. “E” stands for the edge of the exon, while “M” stands for the middle of the exon. Approximately 85% of placenta-specific chimeric RNAs necessitate the 5′ breakpoint to be located at the exon's edge, potentially facilitating the utilization of canonical splicing sites (Fig. S1B). Considering the less significance and low validation rate of the chimeric RNAs spliced between the middle of parental gene exons, the “MM” category of chimeric RNAs was filtered out.

**Figure 1:**
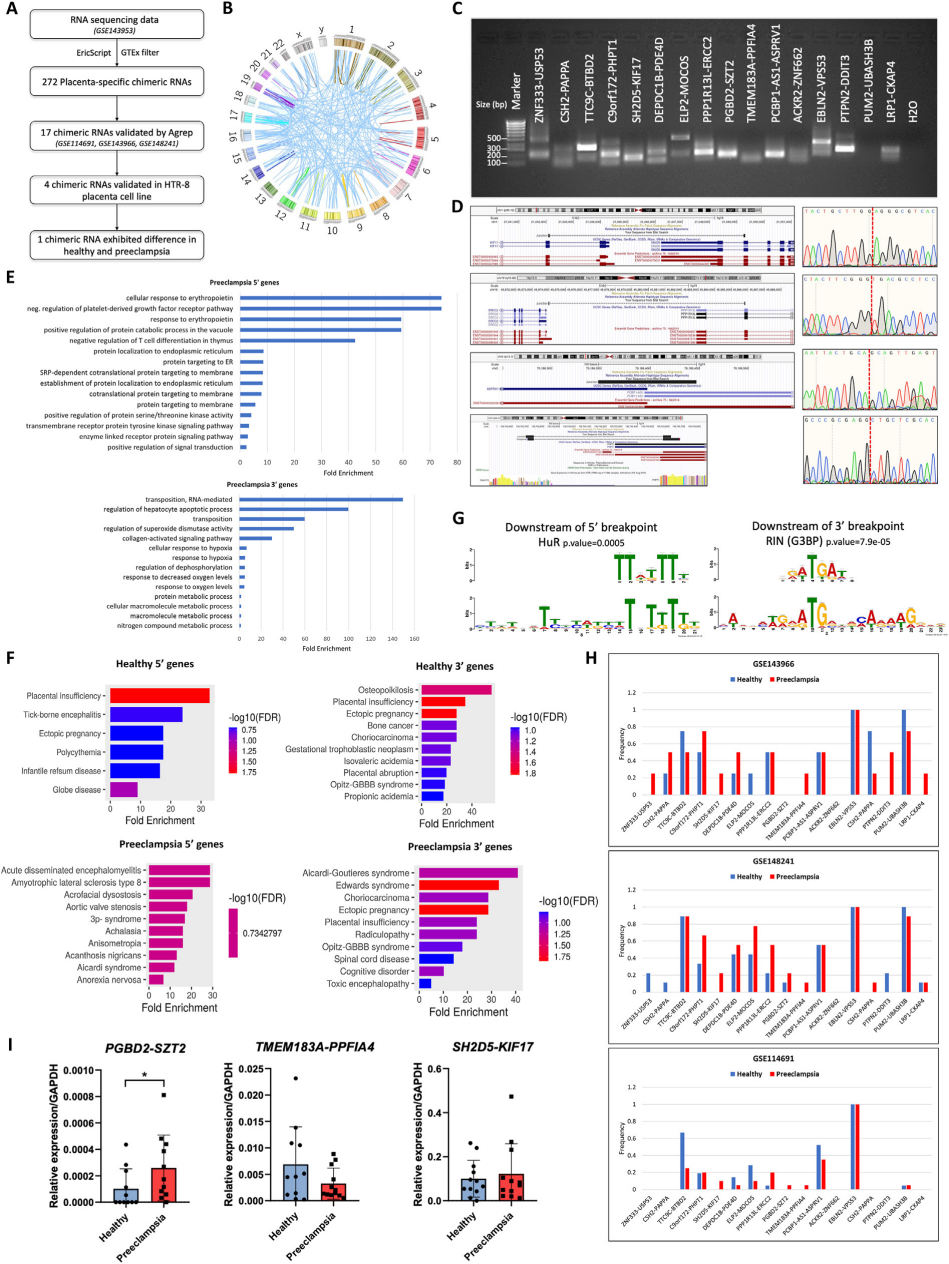
The landscape of chimeric RNAs in healthy and preeclampsia placentas. **(A)** The pipeline for the identification and validation of chimeric RNAs in the placenta. EricScript software was used to identify chimeric RNAs from placenta RNA-sequencing data. Bioinformatic and experimental methods were used to validate chimeric RNA candidates and detect their differential expression between preeclampsia and healthy placenta. **(B)** The Circos plot indicating chimeric RNAs identified from GSE143953 RNA-sequencing database. The lines denote the chimeric RNAs connecting two parental genes. **(C)** Gel electrophoreses of the PCR products of 17 candidate chimeric RNAs. **(D)** Sanger sequencing results of the validated chimeric RNAs. The PCR-amplified junction sequences were aligned against the human genome using BLAT. The alignment results were visualized in the UCSC browser. The red dotted line indicates the breakpoint of the chimeric RNAs. **(E)** Gene Ontology (GO) analysis of the 5′ and 3′ parental genes involved in chimeric RNAs identified in preeclampsia placenta. The signaling pathways enriched for the listed genes were displayed with fold enrichment values. **(F)** Gene enrichment analysis was performed against the JENUS Diseases databases. **(G)** Enriched sequences and RNA binding motifs at the breakpoints of chimeric RNAs identified in preeclampsia placenta. The lower panel indicates the enriched sequences predicted by GLAM2 and the upper panel indicates the enriched RNA binding motifs analyzed by Tomtom. No significant binding proteins were found upstream of the 5′ or 3′ breakpoint. **(H)** The Agrep results indicate the frequency of 17 candidate chimeric RNAs compared between preeclampsia and healthy placenta samples in three databases. The frequency of chimeric RNAs is represented by the match counts. **(I)** Quantitative reverse transcription-PCR validation of chimeric transcripts *PGBD2-SZT2*, *TMEM183A-PPFIA4*, and *SH2D5-KIF17* in clinical placenta tissues, including 12 preeclampsia patients and 12 healthy pregnant women. *GAPDH* transcript levels were used as controls for normalization.

To validate the chimeric RNAs predicted by EricScript, we used Agrep, a string-matching tool, to detect chimeric RNAs by finding sequences from the RNA-seq database that match the expected pattern of junction sequences. We obtained 28 nt of junction sequence identified by EricScript as input and validated the chimeric RNAs in three publicly available placenta RNA-sequencing databases (GSE114691, GSE143966, and GSE148241). Ultimately, we detected 17 chimeric RNAs by Agrep in at least one of the databases (Table S1).

To further validate the candidate chimeric RNAs, we utilized reverse transcription-PCR to detect these 17 placenta-specific chimeric RNA candidates in the HTR-8 human trophoblast cell line. The PCR products amplified using primers designed to flank the junction sites, were visualized on an agarose gel (Fig. 1C). To confirm the accuracy of the junction sequence, Sanger sequencing was performed on the DNA samples extracted from the gel. Four chimeric RNAs *SH2D5-KIF17*, *PPP1R13L-ERCC2*, *PCBP1-AS1-ASPRV1*, and *C9orf172-PHPT1* were successfully validated, with the junction sequences matching the predictions (Fig. 1D). Even though a subset of predicted chimeric RNAs may not necessarily be expressed in the trophoblast cell line, the successfully validated chimeric RNAs support the feasibility of the chimeric RNA identification pipeline and uncover a novel reservoir of chimeric RNAs specifically expressed in placenta.

To understand the potential mechanisms and functions of the chimeric RNAs in the placenta and to compare their differences between preeclampsia patients and normal pregnant women, we downloaded the public database GSE143953 which includes placental samples of four preeclampsia and four control patients and analyzed the chimeric RNA profiles in these samples. From the 272 placenta-specific chimeric RNAs, 120 were predicted to be exclusively expressed in preeclampsia placenta, while 127 were predicted to be exclusively expressed in healthy placenta. To profile the characteristics of the chimeric RNAs, we first investigated the chromosome distribution of parental genes involved in chimeric RNA formation. In healthy placenta, parental genes of the predicted chimeric RNAs were significantly enriched on chromosome 7. While in preeclampsia placenta, there was no significant distribution of parental genes involved in the predicted chimeric RNAs (Fig. S2).

Preeclampsia is a complex pregnancy-related disorder characterized by elevated blood pressure and the presence of proteinuria after 20 weeks of gestation.^3^ Some other symptoms also present with the condition such as severe headaches, visual disturbances, shortness of breath, liver failure, accelerated erythropoiesis, and decreased levels of platelets.^4^ To gain deeper insights into the functional implications of the chimeric RNAs identified in preeclampsia placenta, we performed gene ontology (GO) of their parental genes. Among the enriched biological processes associated with the 5′ parental genes, several key pathways were identified, notably involving responses to erythropoietin and the negative regulation of platelet-derived growth factor receptors (Fig. 1E). Interestingly, studies have shown that anemia is a predisposing factor of severe preeclampsia. The enrichment of 5′ genes associated with chimeric RNAs in preeclampsia within the cellular response to the erythropoietin pathway suggests the potential role of chimeric RNAs in regulating erythropoiesis in preeclampsia. On the other hand, the 3′ parental genes exhibited significant correlations with the regulation of hepatocyte apoptotic process and oxygen metabolism pathways (Fig. 1E). These findings suggest potential roles of the chimeric RNAs in blood pressure regulation, liver function, and oxygen utilization. The identification of these pathway associations provides insights into the functional implications of the chimeric RNAs in preeclampsia pathology. We then performed the enrichment analysis to study the disease correlations of the parental genes involved in chimeric RNAs. In the healthy placenta, both the 5′ and 3′ parental genes of chimeric RNAs were significantly correlated with placental insufficiency, highlighting the potential function of chimeric RNAs in placental development (Fig. 1F). However, the disease correlations of chimeric RNAs in preeclampsia placenta were more diverse and complex. The 5′ and 3′ parental genes of these chimeric RNAs showed associations with various diseases, including acute disseminated encephalomyelitis, amyotrophic lateral sclerosis, Aicardi Goutières syndrome, and choriocarcinoma (Fig. 1F). Their involvement in pathways related to neurodegenerative diseases and cancer indicates their cross-talk with disease-related pathways in preeclampsia.

To investigate the potential mechanisms underlying chimeric RNA formation, we aim to identify potential proteins that might bind at the junction sites of the chimeric RNAs. Initially, we detected enriched sequences at the breakpoint and subsequently applied the Tomtom tool to identify RNA binding motifs enriched in these sequences. In the healthy placenta, canonical splicing factors such as SRSF10, IGF2BP2, YBX2, and SYNCRIP were predicted to bind to the chimeric RNAs at the junction sites (Fig. S3). Remarkably, in preeclampsia placenta, protein HuR was predicted to bind downstream of the 5′ breakpoint and RIN was predicted to bind downstream of the 3′ breakpoint (Fig. 1G). No significant binding proteins were found upstream of the 5′ or 3′ breakpoint. It has been reported that HuR and RIN (also known as G3BP) mediate the assembly of cytoplasmic stress granules in placentas in women with preeclampsia.^5^ These findings suggested the potential mechanisms underlying the formation of chimeric RNAs specific to preeclampsia placenta.

To explore the potential diagnostic biomarkers for preeclampsia, we aimed to analyze the differential expression of the candidate chimeric RNAs between preeclampsia and healthy placentas. Firstly, we used Agrep to compare the frequency of the validated 17 chimeric RNAs between preeclampsia and healthy placenta RNA-seq data from databases GSE114691, GSE143966, and GSE148241 (Fig. 1H). The chimeric transcripts *PGBD2-SZT2*, *TMEM183A-PPFIA4*, and *SH2D5-KIF17* were specifically detected in preeclampsia samples in GSE114691 database. Their trend of higher expression in preeclampsia samples was consistently observed across all three databases. To further confirm their differential expression between preeclampsia and healthy placenta, we conducted quantitative reverse transcription-PCR-based experimental validation of the three candidate chimeric RNAs in placenta tissues collected from 12 preeclampsia patients and 12 pregnant women at Tongji Hospital. Among them, the chimeric transcript *PGBD2-SZT2* exhibited a significantly higher expression level in preeclampsia placenta (Fig. 1I). Out of the 12 preeclampsia samples examined, 11 tested positive for this chimeric transcript, indicating a high sensitivity rate of 91.7%. This result underscores the potential of *PGBD2-SZT2* as a promising candidate biomarker for preeclampsia. In our future investigations, we aim to delve deeper into exploring both the potential function and underlying mechanism of PGBD2-SZT2 in the context of preeclampsia.

In summary, our research uncovers a novel reservoir of chimeric RNAs specifically expressed in placenta tissues and provides initial evidence supporting their potential roles in preeclampsia pathogenesis. The comprehensive studies exploring the functions and mechanisms of chimeric RNAs will deepen our insights into placental biology and contribute to the development of innovative diagnostic and therapeutic approaches for preeclampsia and other pregnancy complications. In the future, we aim to identify pregnancy-specific chimeric RNAs present in circulating blood, which will allow us to uncover novel diagnostic markers for various gestational complications.

## Ethics declaration

The study was reviewed and approved by the Institutional Review Board of Tongji Hospital, Tongji Medical College, Huazhong University of Science and Technology, China (TJ-IRB20231286). Informed consent was obtained from all subjects involved in the study. Written informed consent has been obtained from all enrolled patients to publish this paper.

## Author contributions

J.X. and H.L. conceived the idea; S.S., S.H., and N.H. performed bioinformatic analysis; X.S., N.H., T.M., and C.P. performed experimental validation; T.M. and C.P. performed Agrep analysis; J.X., S.L., and P.W. collected the clinical samples and performed experimental validation; X.S. and H.L. interpreted the data; X.S., J.X., and H.L. wrote the manuscript; H.L. and P.W. reviewed the data and the manuscript. All authors read the final version of the manuscript and agreed to its publication on Genes & Diseases.

## Conflict of interests

The authors declare that they have no conflict of interests.

## Funding

J.X. received a fund from the Chinese Study Council for the studying abroad program.

## Data availability

The authors confirm that the data supporting the findings of this study are available within the article and its supplementary materials. RNA-seq data are publicly available in the GEO database (accession numbers: GSE143953, GSE114691, GSE143966, and GSE148241).
