# Supplementary material for: Chimeric RNA landscape in the placenta: A transcriptomic analysis revealing novel diagnostic biomarkers forpreeclampsia
Source: Genes Dis. 2024 Feb 9;12(1):101242. doi: 10.1016/j.gendis.2024.101242 (PMC11471197; doi:10.1016/j.gendis.2024.101242)
Supplement: Multimedia component 1 [file mmc1.docx]

**Figure S1** Distributions of identified placenta-specific chimeric RNAs.


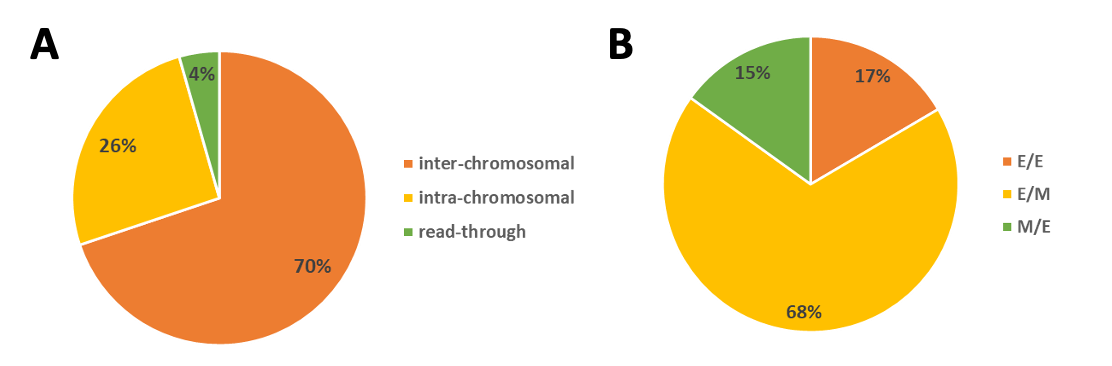


**Figure S2** Chromosome distributions of parental genes involved in placenta-specific chimeric RNAs.


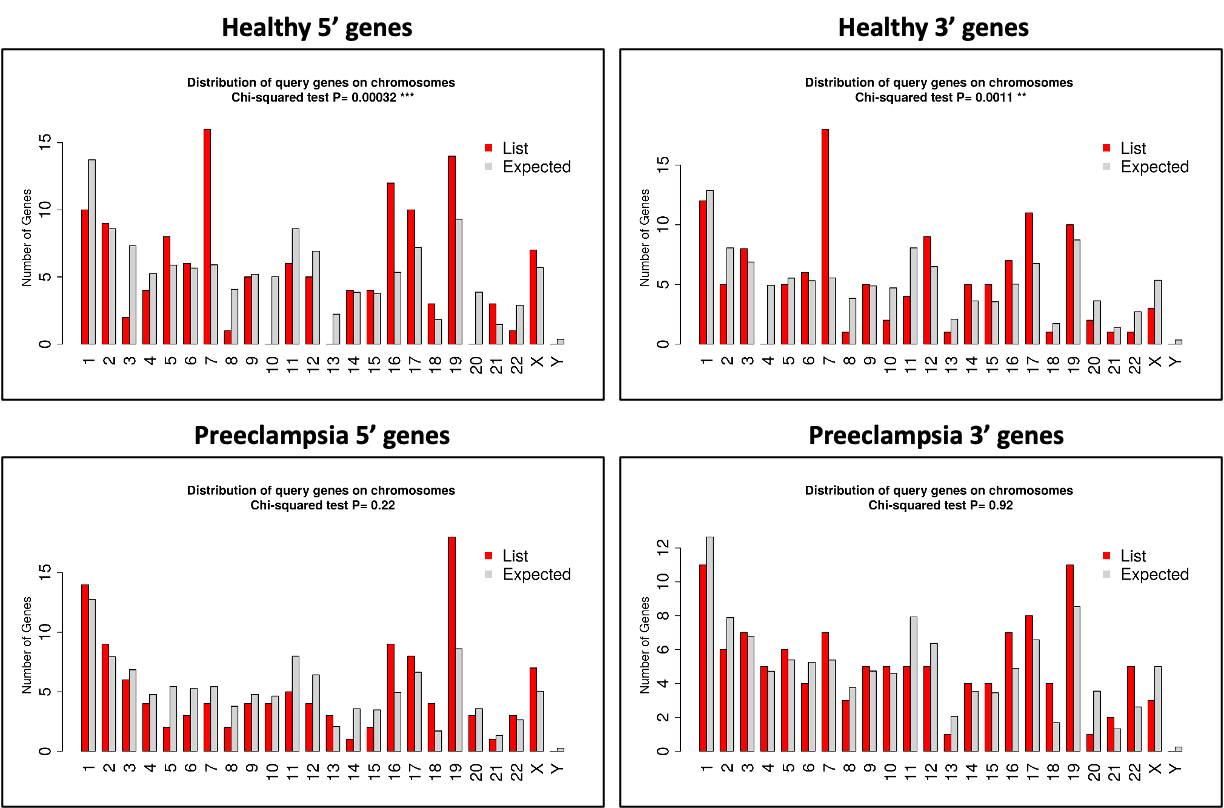


**Figure S3** Prediction of the RNA binding motifs at the breakpoint of chimeric RNAs identified in the healthy placenta.


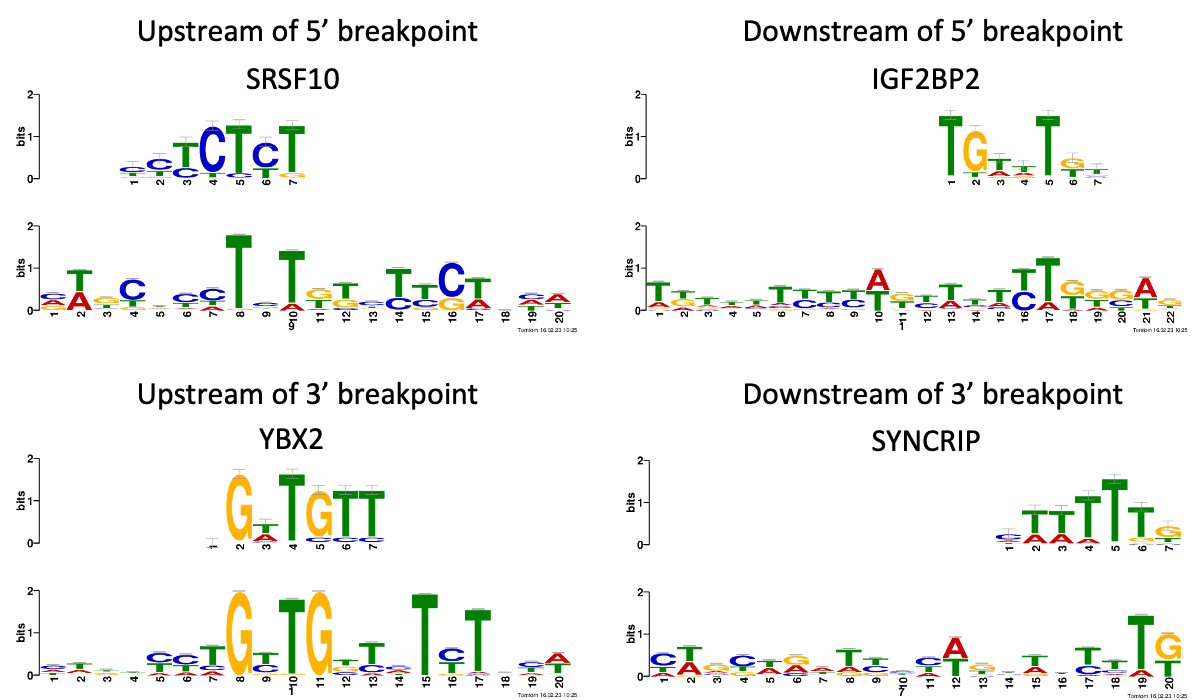


**Table S1** Candidate chimeric RNAs identified by Agrep in placenta RNA-sequencing databases.


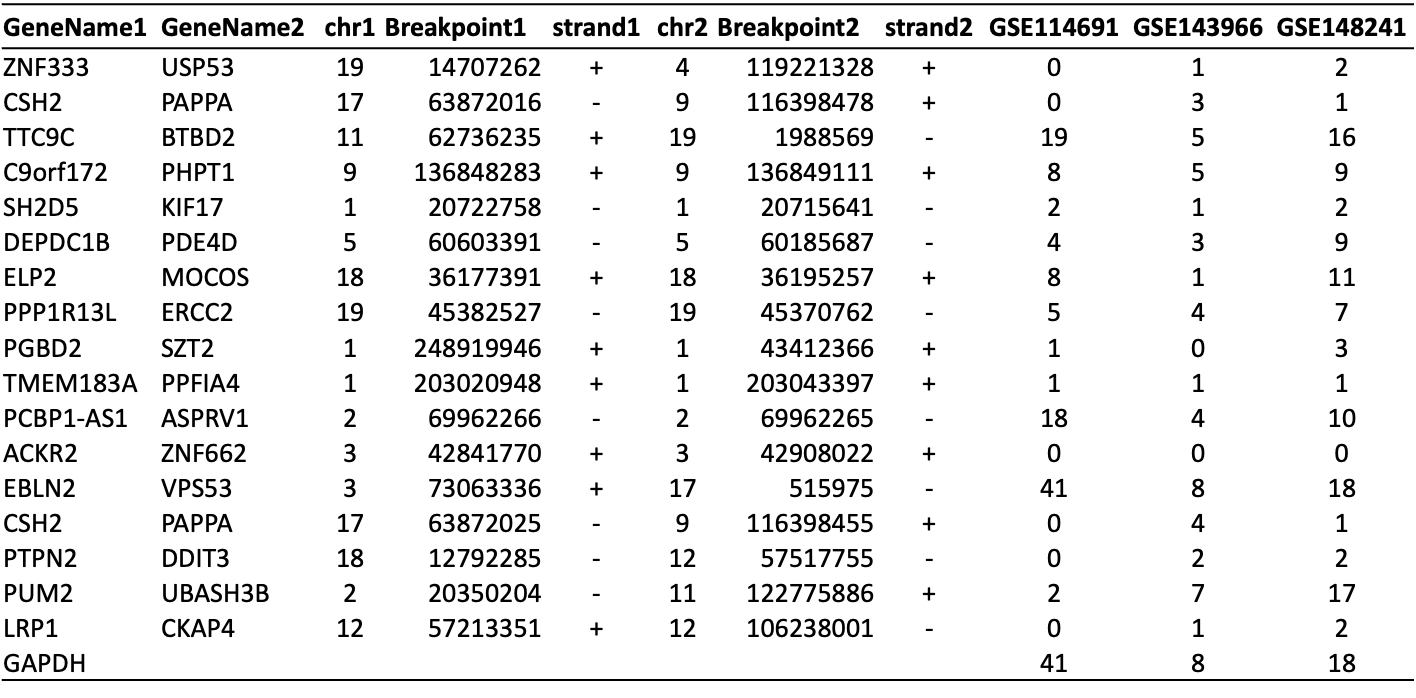


**Table S2** Primers used for chimeric RNAs validation.

| **GeneName 1** | **GeneName 2** | **Forward Sequence 5'-3'** | **Reverse Sequence 5'-3'** |
| --- | --- | --- | --- |
| ZNF333 | USP53 | TGAGGCCAGGAGTTCAAGAC | CACTGCAACCTTGACCTCCT |
| CSH2 | PAPPA | GGTAGCTGTGGCTTCTAGGTG | TTGATCACCAGGTCAGTGCT |
| TTC9C | BTBD2 | AGGCAGGAGAATTGCTTGAA | TGCCTGCCTGAGACCTAATC |
| C9orf172 | PHPT1 | CCAGGTGGGAGCCTGGAG | CAATCGCAATCCAGTTCCTT |
| SH2D5 | KIF17 | GGCCAGTGGTGTCTGTCC | CCCTGCATGGTGAAGGAC |
| DEPDC1B | PDE4D | TTGGCCTTGCAGACCTCTAT | TGATCTCACTGCACGTATCCA |
| ELP2 | MOCOS | CACGGCACCATTATTCACAG | CACCTGCTCCACAGTGTCAT |
| PPP1R13L | ERCC2 | GGAGACCGACTGGTGGTG | CGAGACCATTGGCTAACCTG |
| PGBD2 | SZT2 | TGTTGATCAGTGATGTTGAGCA | ACAGGAAGCGGACAGACAGT |
| TMEM183A | PPFIA4 | ACCAGGACTGCTGCCTACTG | CGTGTGTTATTCCGTTCTGC |
| PCBP1-AS1 | ASPRV1 | ACAATGCTCATGGAGTGCAG | CACACACACACGTGCACAAT |
| ACKR2 | ZNF662 | GGAATAAGATGCAGCCTTGC | CAGAGAAGTAGACGGCCACA |
| EBLN2 | VPS53 | CAGGATCAACCATCACACTCC | ACCTCGGCTCACTGCAAC |
| CSH2 | PAPPA | GGTAGCTGTGGCTTCTAGGTG | TTGATCACCAGGTCAGTGCT |
| PTPN2 | DDIT3 | GGAGGCTAAGGCAGGAGAAT | TTCAGGTGTGGTGATGTATGAAG |
| PUM2 | UBASH3B | GGTTAGAGACCGAGCTGTCG | GACAGCCCAGGCAGTCTATT |
| LRP1 | CKAP4 | GGCTGGAGACTTCCTCTGGT | ATCCGGCTGACCTCACTCT |
| GAPDH | | CTGACTTCAACAGCGACACC | TTACTCCTTGGAGGCCATGT |

**Materials and Methods**

**Data Acquisition**

The RNA-seq data of healthy individuals were downloaded from the Genotype-Tissue Expression (GTEx) project (V6 dbGaP Accession phs000424.v6.p1). To discover the potential placenta-specific chimeric RNAs, we downloaded the RNA-seq data of the placenta tissue from GSE143953. To explore the differential expression of the candidate chimeric RNAs between the healthy and preeclampsia pregnant women, we used the public databases GSE114691, GSE143966, and GSE148241 which include the RNA-seq data from the placenta tissue of the early-onset preeclampsia and the age-matched healthy individuals.

**Sample collection**

Placenta tissues were collected from 12 preeclampsia patients and 12 aged-matched pregnant women at Tongji Hospital, Chica. Patients with following medical histories before pregnancy were excluded: hypertension, kidney disease, diabetes, systemic lupus erythematosus, or thrombotic disorders. The diagnosis of preeclampsia was confirmed in patients exhibiting high blood pressure readings of ≥140/90 mmHg and urinary protein levels of ≥ 0.3g/24h during the gestational period between 36 and 39 weeks. The RNA samples extracted from the placentas must meet purity criteria, with 260/280 ≥ 1.6 and 260/230 ≥ 1.8.

**Bioinformatic Prediction of Chimeric RNAs**

We used the software tool EricScript with default parameters to identify chimeric RNAs. The GRCh38 human reference genome and the paired-end RNA-seq fastq files were used as input. Identified chimeric RNAs with the EricScore more than 0.6 were selected. Blat was used to apply a sequence identity-based filter to remove potential false positive chimeras. Based on the junction coordinates, chimeric RNAs were classified into “E/E”, “E/M”, “M/E”, or “M/M” (“E” and “M” represent that the junction site of 5’ and 3’ gene are located at the edge or middle of exons).

**AGREP**

Using the junction sequence provided by EricScript, we utilized a 28nt junction sequence with 14nt from each side as input into the agrep string-matching software package. No error of mismatch between the 28nt junction sequence and the raw sequence was allowed. The frequency of the chimeric RNA was represented by the match counts detected from agrep. Fisher’s exact test was used to compare the statistical significance between 38 healthy and 38 preeclampsia samples. p-values < 0.05 were considered statistically significant.

**qRT-PCR and Sanger sequencing**

Candidate chimeric RNAs were validated by real-time RT-PCR. RNA samples were extracted by TRIzol from the human trophoblast cell line HTR-8. cDNA library was generated by the Verso cDNA synthesis kit. Specific primer pairs for the candidate chimeric RNAs were designed at 50-150bp upstream or downstream of the junction site. All primers used for validation are listed in Table S2. Real-time PCR was performed with SYBR green dye. Gel electrophoresis was then performed, and the desired DNA band was extracted for Sanger sequencing. To confirm the junction sequence of the validated chimeric RNA, the raw sequence from Sanger sequencing was aligned to the GRCh38 human reference genome through the BLAT tool in the UCSC browser.

RNA was reverse-transcribed via random primers. SYBR green-based qRT-PCR was performed. Relative RNA levels were calculated using the ΔΔCt method. Chimeric RNA expression was normalized to the housekeeping gene GAPDH. Student T test was used to compare the statistical significance between the healthy and preeclampsia samples. p-values < 0.05 were considered statistically significant.

**Gene Ontology and Enrichment Analysis**

Gene ontology terms were predicted for the 5’ and 3’ parental genes of chimeric RNAs by using GOrilla (https://cbl-gorilla.cs.technion.ac.il). All annotated genes in GRCh38 were used as the enrichment background. The distribution of 5’ and 3’ parental genes on chromosomes and their functional enrichment were analyzed by using ShinyGO v0.77 (<http://bioinformatics.sdstate.edu/go/>). Jensen Diseases database was used to study the disease-gene associations.

**RNA Binding Motif Analysis**

Gapped Local Alignment of Motifs (GLAM2) tool from MEME SUITE was used with default parameters to identify enriched motifs. By using GLAM2, we assessed the 5’ and 3’ parental gene sequences with 200 base pairs upstream and downstream of the chimeric junctions to determine enriched motifs. Tomtom tool from MEME SUITE was used with default parameters, along with a known RNA binding protein motifs database, to determine the enriched motifs associated RNA binding proteins.
